# Retrospective assessment of neoadjuvant camrelizumab combined with induction chemotherapy: efficacy in laryngeal preservation for advanced hypopharyngeal and laryngeal squamous cell carcinoma

**DOI:** 10.1007/s00262-023-03579-0

**Published:** 2024-02-15

**Authors:** Jin Peng, Guangfeng Luo, Yongchao Yu, Kang Ning, Xuekui Liu

**Affiliations:** State Key Laboratory of Oncology in South China, Guangdong Provincial Clinical Research Center for Cancer, Sun Yat-Sen University Cancer Center, Guangzhou, 510060 China

**Keywords:** Hypopharyngeal cancer, Laryngeal cancer, Squamous cell carcinoma, Camrelizumab, Immunotherapy, Laryngeal preservation

## Abstract

**Background:**

Hypopharyngeal and laryngeal squamous cell carcinoma (SCC) account for 25–30% of head and neck SCC. Total laryngectomy, while effective, compromises the quality of life. Immune checkpoint inhibitors such as Camrelizumab offer potential in laryngeal preservation. The study investigated Camrelizumab combined with TP regimen as a neoadjuvant therapy for laryngeal preservation in advanced hypopharyngeal and laryngeal SCC.

**Methods:**

A retrospective study was conducted at Sun Yat-sen University Cancer Center on patients diagnosed with locally advanced SCC of the hypopharynx and larynx from October 1, 2019, to October 25, 2022. The efficacy of a first-line treatment combining Camrelizumab (200 mg) and TP regimen (Albumin-bound paclitaxel at 260 mg/m^2^ and Cisplatin at 60 mg/m^2^) was evaluated using RECIST 1.1 criteria. Outcomes included overall survival (OS), progression-free survival (PFS), laryngectomy-free survival (LFS), and response rates.

**Results:**

Of the 71 included patients, the median age was 60.7 years. Post the first-line treatment, 90.1% demonstrated an overall response. The one-year and two-year OS rates were 91.5% and 84.3%, respectively. One-year and two-year PFS rates were 92.9% and 83.9%, respectively, with LFS at 85.6% and 73.2%. The initial T4 stage as significantly associated with reduced OS and LFS. Skin reaction was the predominant adverse event.

**Conclusion:**

The Camrelizumab-TP regimen demonstrated promising results for advanced hypopharyngeal/laryngeal SCC patients, exhibiting high response rates, OS, and LFS, positioning it as a potential primary option for laryngeal preservation. Further comprehensive, randomized controlled studies are imperative to validate these initial observations and elucidate the regimen's full clinical efficacy in optimizing laryngeal outcomes.

**Supplementary Information:**

The online version contains supplementary material available at 10.1007/s00262-023-03579-0.

## Introduction

Laryngeal and hypopharyngeal squamous cell carcinoma (SCC) represent a substantial proportion of head and neck squamous cell carcinoma (HNSCC) cases, constituting an estimated 25–30% of the total [1, 2]. While total laryngectomy offers a definitive treatment approach, it significantly compromises patients' quality of life due to the larynx's pivotal roles in swallowing, vocalization, and airway protection [3, 4]. Consequently, for those with locally advanced disease, the emphasis has shifted toward neoadjuvant therapies that prioritize laryngeal preservation [5].

Prior to the 1990s, advanced-stage laryngeal cancer was predominantly treated with total laryngectomy. This landscape shifted post the 1990 VA study which advocated for nonsurgical organ preservation using induction chemotherapy [6]. This transition was fine-tuned by radiation therapy oncology group (RTOG) study 91–11 which promoted concurrent chemoradiation. This method favored laryngeal preservation, but with a suboptimal long-term overall survival (OS): a 5-year rate of 55% and a 10-year at 28%, potentially due to treatment toxicity [7]. Notably, key studies often overlooked advanced-stage T4 patients, resulting in a paucity of data for laryngeal preservation in this cohort. Comparative analyses showed negligible survival difference between non-surgical larynx preservation and total laryngectomy for stage III cases with low lymph node load. However, T4 cases, or those with a significant lymph node burden, might lean toward total laryngectomy [8]. Another study suggested that laryngeal preservation may slightly curtail 2- and 5-year OS rates for stage III/IV patients versus surgery [9]. With the abundance of therapeutic options, the DeLOS-II trial explored Cetuximab's addition to induction chemotherapy and radiotherapy for locally advanced laryngeal/hypopharyngeal cancers. However, the 24-month laryngectomy-free survival (LFS) and OS were unremarkable [10]. This emphasizes the dire need for enhanced strategies to substantiate laryngeal preservation evidence in advanced-stage patients.

Over the past decade, Immune checkpoint inhibitors (ICIs) have revolutionized advanced cancer treatment, transitioning from second or third-line to first-line agents [11]. Among them, anti-PD-1 inhibitors, designed to engage cell-surface immune checkpoints, counteract immune inhibitory pathways [12]. Given the overexpression of their ligand, PD-L1, in many HNSCC cells, these drugs have garnered FDA approval for use in this patient subset [13–15].

Camrelizumab, an anti-PD-1 monoclonal antibody (mAb), has shown promise in various malignancies [16, 17]. In combination with chemotherapy for locally advanced HNSCC, it achieved high response rates of 96.7% (29/30) [18]. When paired with the VEGFR2 inhibitor Lapatinib, it yielded a 40% major pathological response (defined as ≤ 10% residual viable tumor cells) in advanced oral SCC, especially in patients with higher PD-L1 scores [19]. For unresectable hepatocellular carcinoma, Camrelizumab plus Apatinib surpassed sorafenib in progression-free and overall survival [20]. In refractory nasopharyngeal carcinoma, Camrelizumab combined with Apatinib demonstrated significant antitumor activity, though with notable adverse events [21]. Overall, Camrelizumab’s potential in diverse cancer treatments is evident.

Given the current paucity of clinical studies on Camrelizumab’s application in local advanced hypopharyngeal and laryngeal cancers, this retrospective research aims to investigate the efficacy and laryngeal preservation of the Camrelizumab combined with TP regimen as neoadjuvant therapy, thereby enriching evidence for organ preservation strategies in this subset of patients.

## Methods

### Study design and patient cohort

We conducted a retrospective analysis of patient records from Sun Yat-sen University Cancer Center, a leading tertiary healthcare facility, to identify consecutive individuals who received checkpoint inhibitor (CPI) therapy between October 1, 2019, and October 25, 2022. We focus on patients diagnosed with locally advanced squamous cell carcinoma (SCC) of the hypopharynx and larynx via histopathological or cytological verification.

The objective of our study was to assess the efficacy of Camrelizumab as an initial immunotherapy for larynx preservation. Accordingly, we concentrated our inclusion criteria on individuals aged 18 years and above who were histopathologically diagnosed with squamous cell carcinoma in either the hypopharynx or larynx and had undergone at least three cycles of neoadjuvant therapy, consisting of Camrelizumab monoclonal antibody in combination with albumin-bound paclitaxel and cisplatin (TP regimen). Furthermore, following the neoadjuvant therapy, candidates selected either surgery or concurrent chemoradiotherapy (CCR), contingent upon the degree of lesion remission observed. Additionally, those considered for inclusion maintained complete medical records. On the other hand, exclusion criteria encompassed patients with a non-squamous cell carcinoma pathological diagnosis, those identified with distant metastases prior to initiating treatment, individuals presenting with concurrent malignant tumors or possessing a pertinent medical history, and those having undergone chemotherapy, radiotherapy, or immunotherapy in their medical history.

By delving into the electronic health records, we collated patient-specific information, including age, sex, tobacco use history, Eastern Cooperative Oncology Group (ECOG) performance metrics (scores range from 0 to 5, with higher scores indicating worse functional status), TNM categorization, subsequent therapeutic interventions, CPI therapy specifics, reaction and adverse effect details, dates of laryngectomy surgeries, and either the date of demise or the most recent follow-up. The efficacy of the Camrelizumab + TP treatment strategy was assessed as per the Response Evaluation Criteria in Solid Tumors (RECIST) version 1.1. The Sun Yat-sen University Cancer Center's institutional review board granted approval for this research and deemed informed consent unnecessary due to its retrospective nature. This research aligns with the Strengthening the Reporting of Observational Studies in Epidemiology (STROBE) guidelines.

### Statistical analysis

We performed all statistical computations using SPSS software, version 25.0 (IBM Corp., Armonk, NY, USA). The period from the start of neoadjuvant Camrelizumab + TP therapy to the event of death from any reason was denoted as overall survival (OS). Progression-free survival (PFS) was marked as the interval from the onset of CPI intervention to either radiological evidence of disease advancement or death. Laryngectomy-free survival (LFS) was defined as the duration from the initiation of therapy to the occurrence of objective surgical indications for laryngectomy, undergoing complete or partial laryngectomy, or mortality [10]. We determined the median values for OS and PFS through the Kaplan–Meier estimation techniques. We further utilized both univariate and multivariate Cox proportional hazards models to compute hazard ratios (HRs) and their respective 95% confidence (CI) boundaries for key variables. These encompassed factors such as diagnostic age, sex, primary lesion site, ECOG functional status, clinical categorization, tobacco use, alcohol consumption, and TNM staging. Radiological outcomes were denoted by the best response noted in subsequent imaging evaluations post-CPI therapy commencement, as derived from radiological interpretations and clinical progress records. Individuals demonstrating full or partial radiological improvements were deemed as responders. Conversely, those manifesting stable conditions, disease escalation, or heterogeneous outcomes (delineated as divergent diminution and escalation across distinct lesions) were labeled as non-responders. To evaluate the differences in response rates across various subgroups, we applied the χ2 statistical analysis. Moreover, through multivariable logistic regression, we examined the refined relationships between essential variables and the observed radiological outcomes. The accuracy of our findings was indicated by 95% CI ranges.

## Results

### Cohort baseline characteristics

**Fig. 1:**
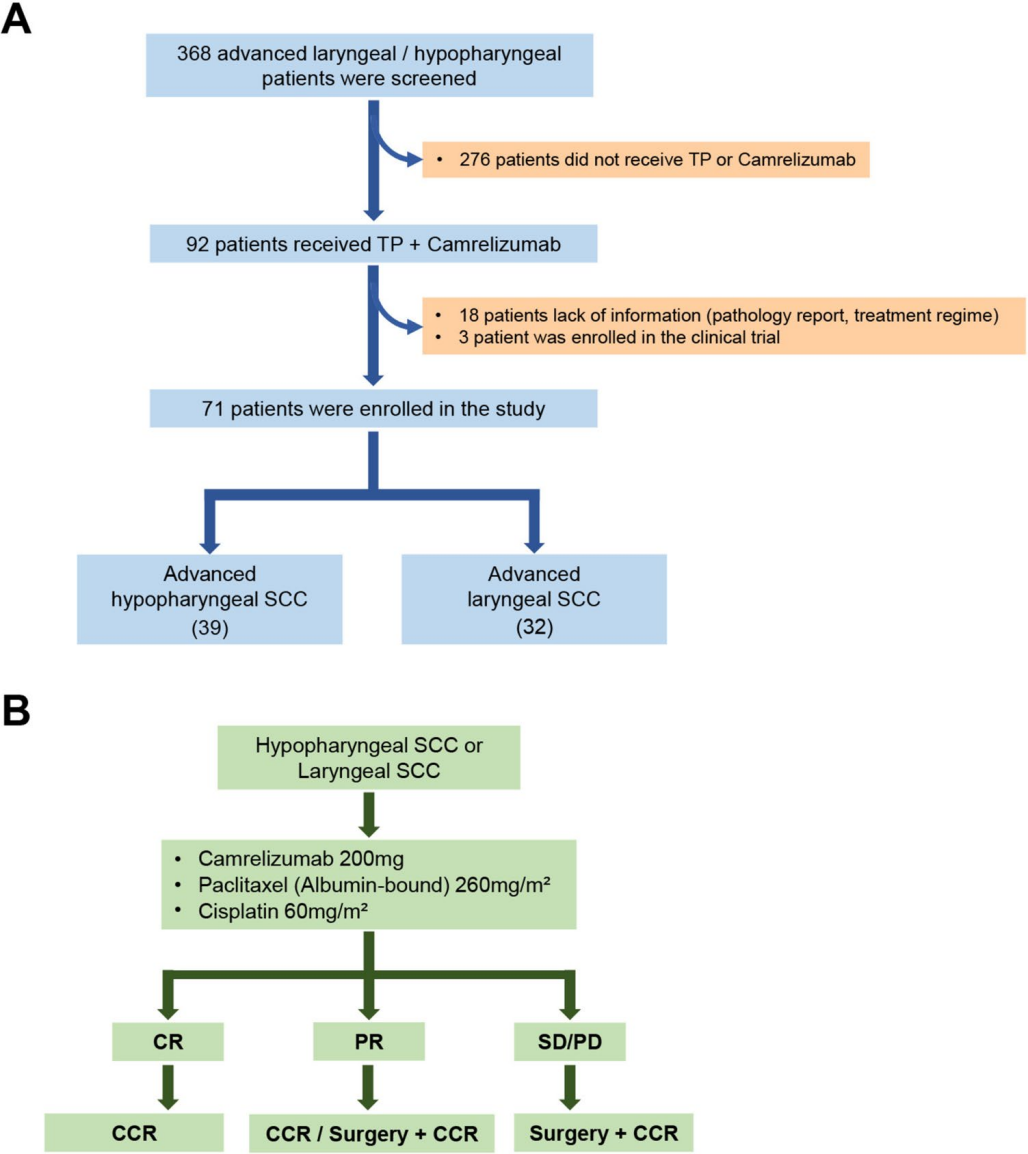
Patient enrollment workflow and treatment trajectory. SCC, squamous cell carcinoma; CR, complete response; PR, partial response; SD, stable disease; PD, progressive disease; CCR, concurrent chemoradiotherapy

The process for patient enrollment is depicted in Fig. 1A. From the initial screening of 92 patients satisfying the specified treatment criteria, 18 patients missing the necessary information and 3 patients involved in a prospective clinical trial of Camrelizumab were excluded. Consequently, 71 patients diagnosed with advanced hypopharyngeal/laryngeal SCC who underwent TP + Camrelizumab as initial therapy were incorporated into the study.

For the 71 patients, the median age at the time of diagnosis was 60.7 years. A significant majority, 67 (94.3%) patients, were male. 13 (18.3%) participants had an ECOG performance score equal to or exceeding two. A notable 56 (78.9%) of these patients presented with clinical stage IV (locally advanced) neoplasms. Smoking was observed in the history or current lifestyle of 24 (33.8%) patients, while 31 (43.7%) had a history or current habit of alcohol consumption (Table 1). Regarding tumor location, the hypopharynx and larynx were predominant, with 39 (54.9%) and 32 (45.1%) instances, respectively. Post TP + Camrelizumab therapy, 60 patients transitioned to CCR, whereas 11 opted for surgical intervention, followed by adjuvant CCR (Fig. 1B).

**Table 1:** Cohort baseline characteristics

Characteristic	Camrelizumab + TP (N = 71)
Age at diagnosis, mean (SD)	60.7 (0.97)
*Sex*	
Male	67
Female	4
*Primary lesion site*	
Hypopharynx	39
Larynx	32
*ECOG functional status*^a^	
0 or 1	58
≥ 2	13
*Initial clinical stage*	
III	15
IV	56
*Tobacco use*	
Non-user	47
Active or Previous	24
*Alcohol consumption*	
Non-user	40
Active or Previous	31
*Initial T-Stage*^b^	
T1	5
T2	24
T3	10
T4	32
*Initial N-Stage*	
N0	15
N1	8
N2	43
N3	5

*SD* Standard Deviation, *ECOG* Eastern Cooperative Oncology Group

^a^Scores oscillate between 0 and 5; higher values denote a compromised functional status

^b^According to the criteria established by the American Joint Committee on Cancer

## Survival and remission following camrelizumab + TP treatment

**Fig. 2:**
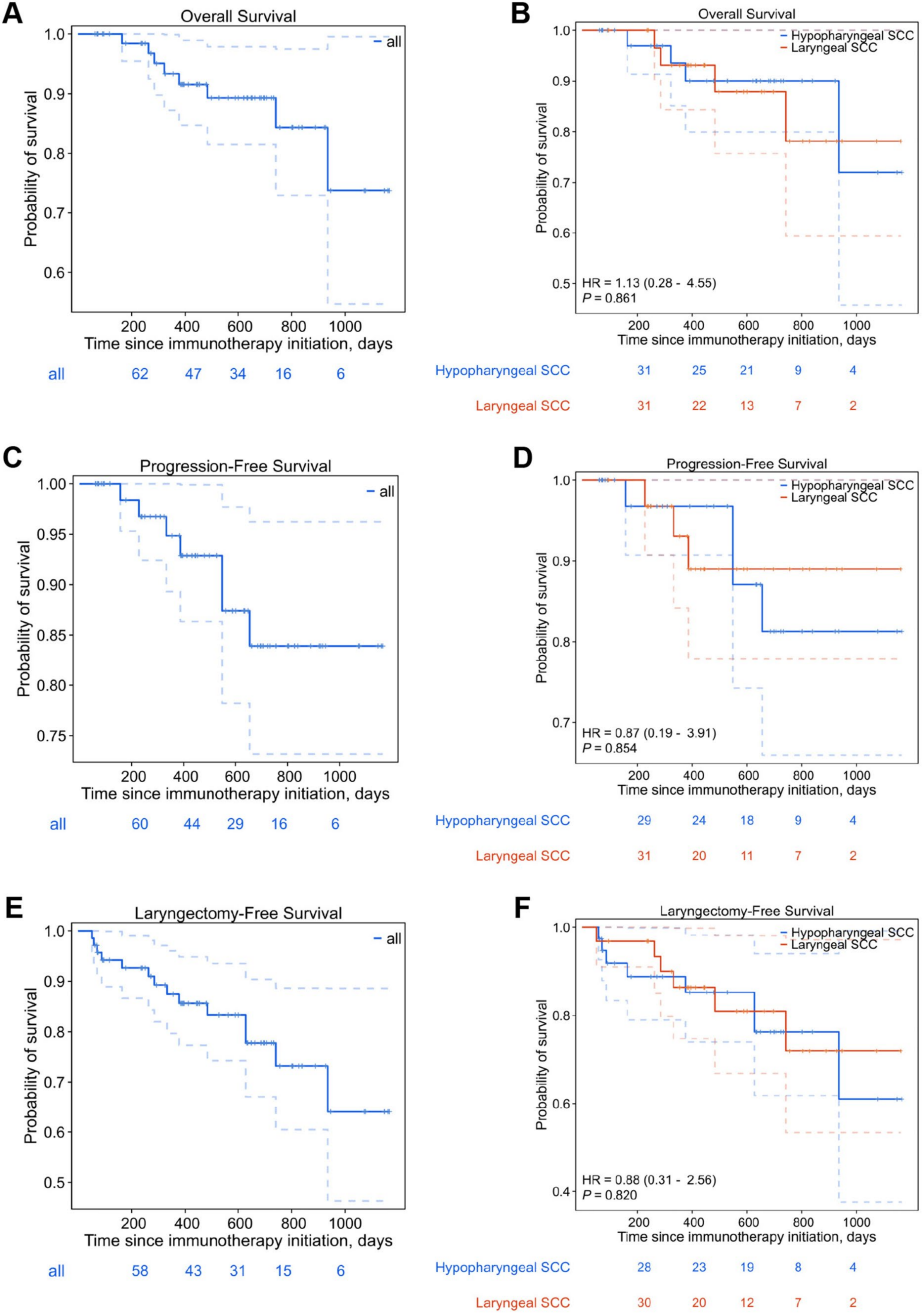
Overall survival (**A**, **B**), progression-free survival (**C**, **D**), and laryngectomy-free survival (**E**,** F**) for the entire cohort and stratified by primary site

The data disclosed a one-year OS rate of 91.5% (95% CI: 84.7%-98.9%) and a two-year rate of 84.3% (95% CI: 73.0%–97.4%). Concurrently, the PFS rate was recorded at 92.9% at one year (95% CI: 86.3%–99.8%) and 83.9% at two years (95% CI: 73.2%–96.2%). Moreover, the one-year and two-year LFS rates were observed to be 85.6% (95% CI: 77.3%–94.8%) and 73.2% (95% CI: 60.4%–88.6%), respectively. A comparative analysis yielded no significant statistical differences in OS, PFS, and LFS between hypopharyngeal and laryngeal SCC (Fig. 2). The cohort was followed up for a median duration of 19.6 months (IQR: 10.8–24.7 months).

Upon conducting a univariate Cox proportional hazards regression, which included covariates such as sex, ECOG score, tobacco and alcohol consumption, primary lesion site, clinical stage, and TNM stages, a significant correlation was identified between decreased OS and T4 category upon initial presentation (HR: 1.817; 95% CI: 1.246–5.810). Additionally, a reduced LFS was linked with the initial T4 category (HR: 1.244; 95% CI: 1.042–2.908). For PFS, the univariate analysis indicated that an absence of cervical lymph node metastasis (stage N0) at diagnosis (HR: 0.471; 95% CI: 0.091–2.431), contributed to enhanced PFS (Table 2). Multivariable assessments did not reveal any significant association with OS, PFS, or LFS (Table 3).

**Table 2:** Univariate Analysis via Cox Proportional Hazards Model for Overall Survival, Progression-Free Survival, Laryngectomy-Free Survival, and Response Rate

		OS		PFS		LFS		Response rate	
Covariate	Total (N)	HR (95% CI)	*P* value	HR (95% CI)	*P* value	HR (95% CI)	*P* value	HR (95% CI)	*P* value
Sex	71								
Male	67	1 [Reference]		1 [Reference]		1 [Reference]		1 [Reference]	
Female	4	0.000 (0.000—Inf)	0.999	0.000 (0.000—Inf)	0.999	0.000 (0.000—Inf)	0.998	2.916 (0.336—25.272)	0.331
*ECOG*^a^	71								
0 or 1	58	1 [Reference]		1 [Reference]		1 [Reference]		1 [Reference]	
≥ 2	13	0.946 (0.114—7.873)	0.959	2.309 (0.442—12.064)	0.321	1.104 (0.243—5.006)	0.898	0.000 (0.000—Inf)	0.998
Tobacco use	71								
Active or previous	24	1 [Reference]		1 [Reference]		1 [Reference]		1 [Reference]	
Non-user	47	1.113 (0.260—4.757)	0.885	1.400 (0.271—7.242)	0.688	1.623 (0.503—5.234)	0.418	0.885 (0.192—4.067)	0.875
Alcohol consumption	71								
Non-user	40	1 [Reference]		1 [Reference]		1 [Reference]		1 [Reference]	
Active or previous	31	1.081 (0.267—4.367)	0.913	1.563 (0.347—7.034)	0.561	1.067 (0.371—3.066)	0.905	0.851 (0.188—3.849)	0.834
Primary lesion site	71								
Hypopharynx	39	1 [Reference]		1 [Reference]		1 [Reference]		1 [Reference]	
Larynx	32	1.196 (0.297—4.811)	0.801	0.848 (0.190—3.793)	0.829	0.844 (0.292—2.442)	0.755	0.496 (0.096—2.560)	0.402
Initial clinical stage	71								
IV	56	1 [Reference]		1 [Reference]		1 [Reference]		1 [Reference]	
III	15	0.329 (0.040—2.723)	0.303	1.084 (0.203—5.781)	0.925	0.590 (0.162—2.151)	0.424	0.000 (0.000—Inf)	0.999
Initial *T*-stage^b^	71								
≤ 3	39	1 [Reference]		1 [Reference]		1 [Reference]		1 [Reference]	
4	32	1.817 (1.246—5.810)	0.042	0.471 (0.091—2.431)	0.369	1.244 (1.042—2.908)	0.041	1.997 (0.432—9.226)	0.376
Initial *N*-stage	71								
≥ 1	56	1 [Reference]		1 [Reference]		1 [Reference]		1 [Reference]	
0	15	0.000 (0.000—Inf)	0.998	0.480 (0.183—0.938)	0.038	0.559 (0.153—2.040)	0.379	0.000 (0.000—Inf)	0.999

^a^Scores oscillate between 0 and 5; higher values denote a compromised functional status

^b^According to the criteria established by the American Joint Committee on Cancer

**Table 3:** Multivariate analysis via cox proportional hazards model for overall survival, progression-free survival, laryngectomy-free survival, and response rate

		OS		PFS		LFS		Response rate	
Covariate	Total (N)	HR (95% CI)	*P* value	HR (95% CI)	*P* value	HR (95% CI)	*P* value	HR (95% CI)	*P* value
Sex	71								
Male	67	1 [Reference]		1 [Reference]		1 [Reference]		1 [Reference]	
Female	4	0.000 (0.000—Inf)	0.999	0.000 (0.000—Inf)	0.999	0.000 (0.000—Inf)	0.998	7.069 (0.294—170.074)	0.228
ECOG^a^	71								
0 or 1	58	1 [Reference]		1 [Reference]		1 [Reference]		1 [Reference]	
≥ 2	13	0.849 (0.079 – 9.151)	0.893	3.830 (0.552 – 26.576)	0.174	1.233 (0.229 – 6.639)	0.808	0.000 (0.000 – Inf)	0.999
Tobacco use	71								
Active or previous	24	1 [Reference]		1 [Reference]		1 [Reference]		1 [Reference]	
Non-user	47	1.491 (0.175—12.713)	0.715	2.670 (0.364—19.584)	0.334	2.848 (0.605—13.399)	0.185	2.099 (0.176—25.060)	0.558
Alcohol consumption	71								
Non-user	40	1 [Reference]		1 [Reference]		1 [Reference]		1 [Reference]	
Active or previous	31	1.357 (0.196—9.403)	0.757	3.319 (0.509—21.660)	0.210	2.017 (0.513—7.937)	0.315	0.456 (0.040—5.153)	0.526
Primary lesion site	71								
Hypopharynx	39	1 [Reference]		1 [Reference]		1 [Reference]		1 [Reference]	
Larynx	32	1.723 (0.333—8.929)	0.517	0.775 (0.133—4.506)	0.777	1.016 (0.297—3.472)	0.98	0.553 (0.105—2.905)	0.484
Initial clinical stage	71								
IV	56	1 [Reference]		1 [Reference]		1 [Reference]		1 [Reference]	
III	15	2.411 (0.172—33.782)	0.513	0.435 (0.013—14.535)	0.642	0.389 (0.030—5.006)	0.469	0.000 (0.000—Inf)	0.999
Initial *T*-stage^b^	71								
≤ 3	39	1 [Reference]		1 [Reference]		1 [Reference]		1 [Reference]	
4	32	0.946 (0.191—4.672)	0.945	0.371 (0.054—2.548)	0.313	0.615 (0.167—2.259)	0.464	0.933 (0.164—5.293)	0.937
Initial *N*-stage	71								
≥ 1	56	1 [Reference]		1 [Reference]		1 [Reference]		1 [Reference]	
0	15	0.000 (0.000—Inf)	0.998	1.122 (0.049—25.459)	0.942	0.797 (0.082—7.788)	0.846	0.000 (0.000—Inf)	0.999

^a^Scores oscillate between 0 and 5; higher values denote a compromised functional status

^b^According to the criteria established by the American Joint Committee on Cancer

**Fig. 3:**
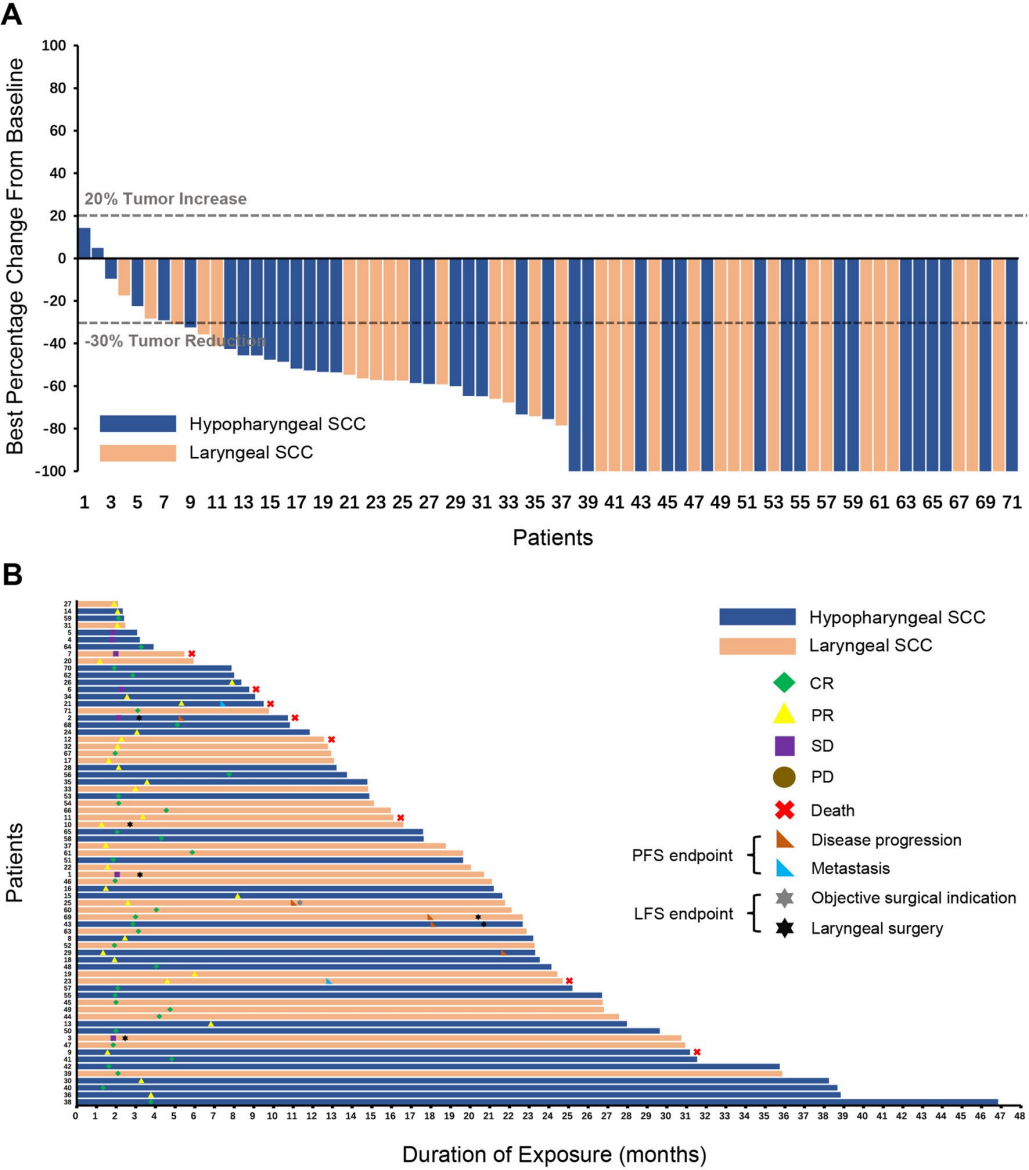
Evaluation of antitumor activity. Analyses were conducted on the efficacy-evaluable population (*n* = 71). **A** Depicts the best percentage change from baseline in target lesion size based on radiographic response. The dashed line at a -30% change serves as the RECIST version 1.1 threshold distinguishing between partial response (PR) and stable disease (SD). **B** Swimlane plot illustrating post-therapy response, progression-free survival, and laryngectomy-free survival for individual participants. CR, complete response; PD, progressive disease

Post Camrelizumab + TP therapeutic intervention, the overall response was determined to be 90.1%. From the 71 patients who had post-treatment imaging data available after the treatment, 34 (47.9%) exhibited a complete response (CR), 30 (42.3%) showed partial response (PR), 7 (9.8%) remained stable disease (SD), with none showing disease progression (PD) (Fig. 3). Neither univariable nor multivariable assessments found any factors significantly impacting the response rate (Table 2 and 3).

**Fig. 4:**
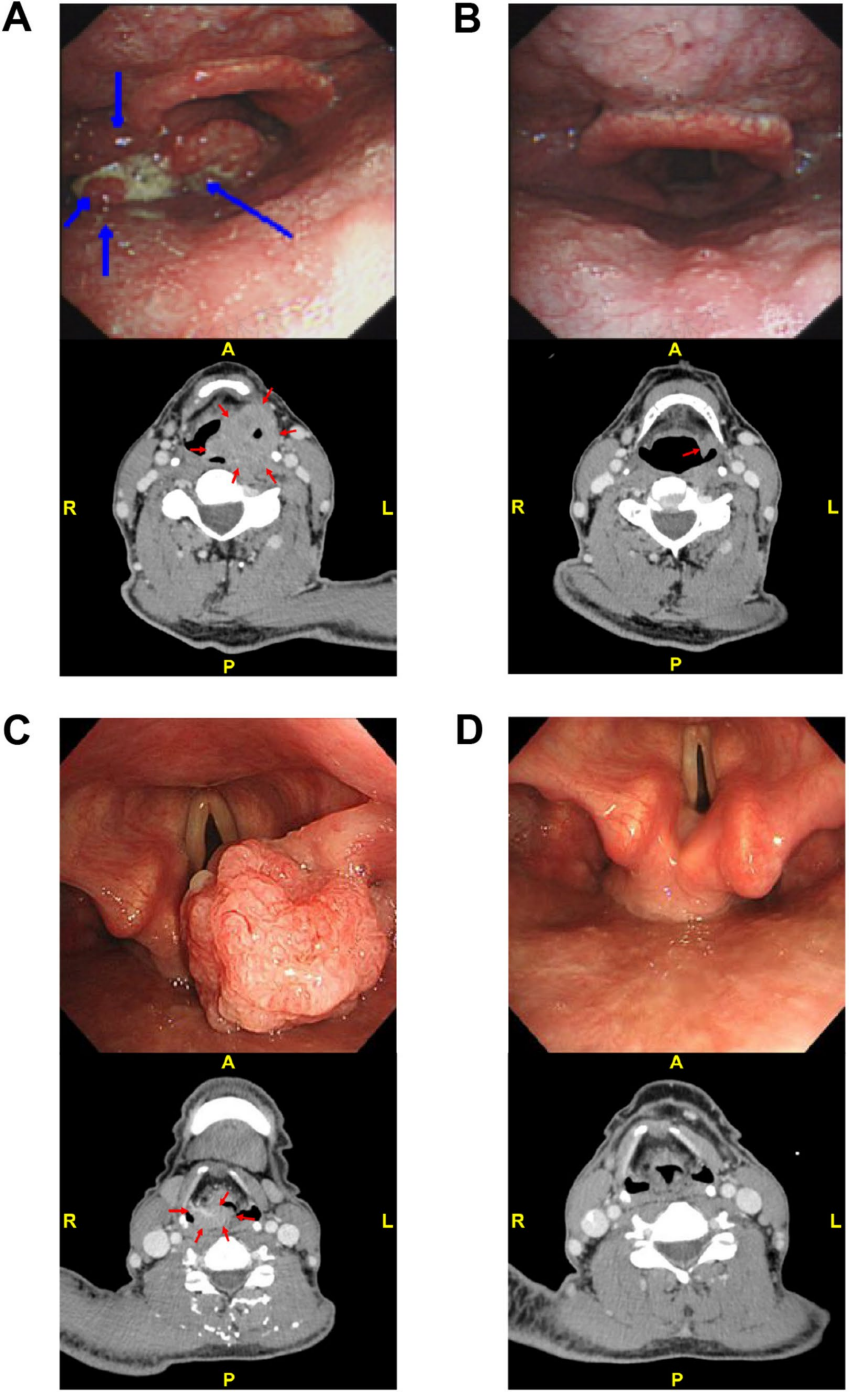
Exemplary Cases of Tumor Regression. **A** Hypopharyngeal SCC (Patient 36, age 62, T4N2bM0, Stage IV); endoscopy and CT identified a left pyriform sinus cT4 SCC. **B** After three cycles of neoadjuvant Camrelizumab + TP therapy, imaging indicated a partial response (PR) of the primary lesion. **C** Supraglottic laryngeal SCC (Patient 56, age 55, T3N2bM0, Stage IV); endoscopy and CT identified a right aryepiglottic fold cT3 SCC. **D** After three cycles of neoadjuvant Camrelizumab + TP therapy, imaging indicated a complete response (CR) of the primary lesion. SCC, squamous cell carcinoma

Figure 4 delineates exemplary tumor regression in two distinct patients. Patient 36, diagnosed with hypopharyngeal SCC, achieved a partial response (PR) evaluation of the lesion after three cycles of Camrelizumab + TP intervention. Patient 56, diagnosed with supraglottic laryngeal SCC, reached a complete response (CR) following four treatment cycles. Notably, throughout the subsequent follow-up period, neither patient experienced events leading to discontinuation of PFS or LFS.

Throughout the treatment phase, 19 patients encountered related adverse reactions (Table 4). The predominant adverse event was skin reactions, affecting 9 patients (47.4%), of which its severity required one patient to receive systemic steroids but without treatment halts. Myelosuppression came second, with 7 instances (36.8%); two patients required blood transfusions and treatment cessation, and resulted in one instance of grade III suppression. The remaining three patients (15.8%) exhibited abnormal thyroid function, which required no further intervention.

## Discussion

The primary focus of this investigation was to assess the efficacy and safety of Camrelizumab + TP as an initial therapeutic strategy for advanced hypopharyngeal/laryngeal SCC. This study incorporated a relatively large cohort of 71 patients, shedding valuable insights on the potential advantages and challenges of this regimen.

At the outset, the baseline characteristics of the study cohort reflected a male predominance (94.3%), which aligns with existing literature suggesting that hypopharyngeal/laryngeal SCC is more commonly diagnosed in males than females. The high incidence of patients presenting at clinical stage IV (78.9%) underscores the relevance of this study to those with advanced-stage neoplasms.

Post Camrelizumab + TP treatment, a promising one-year OS rate of 91.5% and two-year OS rate of 84.3% were observed. Moreover, our one-year PFS and LFS rates of 92.9% and 85.6%, respectively, were encouraging. It is noteworthy that these outcomes did not differ significantly between hypopharyngeal and laryngeal SCC, suggesting a broad applicability of the therapeutic regimen in this subset of HNSCC. In contrast, the DeLOS-II trial with TPF/TP plus cetuximab reported an approximately 69% OS at 24 months [10], and the induction chemotherapy (IC) followed by concurrent chemoradiotherapy (CCRT) study indicated a 3-year OS of 53.1% for the IC + CCRT group [22]. For PFS, our regimen achieved a one-year rate of 92.9% and a two-year rate of 83.9%. Interestingly, the IC + CCRT trial observed no significant PFS difference between its treatment groups, implying no additional advantage of induction chemotherapy. Focusing on larynx preservation, our study outperformed with one-year and two-year LFS rates of 85.6% and 73.2%, respectively. This markedly surpasses the DeLOS-II trial's 24-month LFS of about 47%. Additionally, the IC + CCRT trial emphasized no improvement in larynx preservation with their induction chemotherapy strategy. Collectively, these findings suggest that the Camrelizumab + TP regimen may provide potentially superior or at least comparable superior outcomes of OS, PFS, and LFS. Nonetheless, direct comparisons should be approached with caution given the inherent differences in study designs and populations.

From the perspective of prognostic markers, our univariate analysis divulged a significant relationship between decreased OS and initial T4 categorization, and similarly, a reduced LFS was associated with this category. This implies that patients presenting with T4 categorization might require a more aggressive or diversified therapeutic approach. On the other hand, the absence of cervical lymph node metastasis at diagnosis was linked to a better PFS, reiterating the prognostic significance of nodal status in head and neck cancers. Intriguingly, multivariable analyses did not highlight any factors of significance in association with OS, PFS, or LFS. This might suggest that, while certain variables carry individual prognostic significance, their cumulative impact on survival is multifactorial and possibly moderated by other variables not accounted for in this study.

The overall treatment response rates after Camrelizumab + TP treatment was high at 90.1%, reinforce the therapeutic potential of this regimen. It is noteworthy that nearly half of the cohort (47.9%) exhibited a complete clinical response, pointing toward a substantial subset of patients who might benefit immensely from this therapy.

However, it is imperative to address the adverse events witnessed during the treatment. Skin reaction emerged as the most common adverse event, and while a majority had manageable symptoms, the severity in some instances necessitated the use of systemic steroids. Myelosuppression, the second most common adverse event, led to significant complications for some patients, including the need for blood transfusions and treatment cessation in two cases, and one instance of grade III suppression. These findings necessitate a comprehensive pre-treatment assessment and continuous monitoring throughout the therapeutic course to ensure early identification and management of these adverse events.

The current study provides valuable insights into the efficacy and safety of the TP + Camrelizumab regimen as an initial therapeutic approach for advanced hypopharyngeal/laryngeal SCC. With a fairly sizable cohort of 71 patients, the study offers robust evidence on the potential advantages of this treatment. A notable strength lies in the detailed assessment of survival rates, comprehensive response evaluations, and thorough tracking of adverse events. Furthermore, the incorporation of both univariate and multivariable analyses to identify prognostic factors accentuates the depth of the research, ensuring a multifaceted understanding of patient outcomes. The clear demarcation of baseline characteristics ensures transparency, and the high overall response rate makes the findings noteworthy.

Despite its strengths, the study is not devoid of limitations. Firstly, the research design, which lacks a control or comparative group, might not provide a clear perspective on how Camrelizumab + TP fares against other established treatment regimens. Additionally, the study population showed a pronounced male predominance, which might not allow for extrapolating the findings to a broader, more balanced demographic. Lastly, the median duration of follow-up was less than two years, which might not capture potential long-term outcomes or late-onset adverse events.

## Conclusion

In summary, the Camrelizumab + TP regimen has shown promising therapeutic outcomes in advanced hypopharyngeal/laryngeal SCC. The observed survival rates, in conjunction with the high overall response rates, indicate its potential as a frontline therapeutic strategy. However, the accompanying adverse events underscore the need for meticulous patient evaluation and monitoring. Further multi-centric randomized controlled trials with larger cohorts and diverse demographics will be instrumental in substantiating our findings and establishing the true therapeutic potential of this regimen in advanced hypopharyngeal/laryngeal SCC.

### Supplementary Information

Below is the link to the electronic supplementary material.

Supplementary file1 (DOCX 14 KB)

## Data Availability

Data, analytical procedures, and research materials will be accessible to other investigators upon request directed to the corresponding author.
