# Supplementary material for: Retrospective assessment of neoadjuvant camrelizumab combined with induction chemotherapy: efficacy in laryngeal preservation for advanced hypopharyngeal and laryngeal squamous cell carcinoma
Source: Cancer Immunol Immunother. 2024 Feb 15;73(3):54. doi: 10.1007/s00262-023-03579-0 (PMC10869391; doi:10.1007/s00262-023-03579-0)
Supplement: Supplementary file 1 — Supplementary file1 (DOCX 14 KB) [file 262_2023_3579_MOESM1_ESM.docx]

**Table 4.** Adverse Events

| All (n=19) |  |  |  |
| --- | --- | --- | --- |
| Symptoms | Grade 1-2 | Grade 3-4 | Total |
| Skin reaction | 8 | 1 | 9 (47.4%) |
| Myelosuppression | 6 | 1 | 7 (36.8%) |
| Hypothyroidism | 3 | 0 | 3 (15.8%) |
